# The Challenges of ST-Elevation Myocardial Infarction in COVID-19 Patients

**DOI:** 10.1155/2021/9915650

**Published:** 2021-08-21

**Authors:** Ju Young Bae, Khalil Ian Hussein, Christopher John Howes, John Francis Setaro

**Affiliations:** ^1^Department of Medicine, Greenwich Hospital, Yale-New Haven Health System, 5 Perryridge Road, Greenwich, CT 06830, USA; ^2^Section of Cardiovascular Medicine, Department of Internal Medicine, Yale University School of Medicine, 333 Cedar St, New Haven, CT 06510, USA

## Abstract

By July 2021, the United States had over 34.4 million confirmed COVID-19 cases. Various cardiovascular manifestations of COVID-19 have been reported including ST-elevation myocardial infarction (STEMI), and there is concern that SARS-CoV-2 may be associated with a higher thrombus burden. We performed a retrospective chart review of 535 adult patients with COVID-19 admitted at Yale-New Haven Health Greenwich Hospital from February 1, 2020, to May 13, 2020. All admitted patients had undergone testing for serum troponin I and various inflammatory markers, and we identified three patients who were diagnosed with acute STEMI. Data was collected via manual chart review and included patient demographics, comorbidities, laboratory tests, electrocardiogram (ECG) results, echocardiography results, diagnoses during hospitalization, inpatient therapies, and outcomes including length of hospital stay, revascularization results, and mortality. Three of our patients had obstructive coronary artery disease confirmed via angiography. One subject was noted to display vasospasm in addition to coronary atherosclerotic obstruction and refractory thrombus formation. Among our patients with COVID-19 and STEMI, presentations were variable in terms of timing of onset of ECG changes, age, gender, race, comorbidities, symptomology, and outcomes.

## 1. Introduction

As of July 2021, the United States has over 34.4 million confirmed cases of COVID-19, which is an infectious disease caused by severe acute respiratory syndrome coronavirus 2 (SARS-CoV-2) [1]. SARS-CoV-2 infection has variable manifestations in humans, and there are reported cases of patients with COVID-19 presenting with a broad spectrum of cardiovascular manifestations including acute coronary syndrome, arrhythmia, myopericarditis mimicking ST-elevation myocardial infarction (STEMI), ischemic and stress-induced cardiomyopathy, coronary vasospasm, and pericardial effusion [2–4].

## 2. Case Presentation

We performed a single-center retrospective chart review of 535 adult patients with COVID-19 admitted to our suburban community hospital, Yale-New Haven Health Greenwich Hospital, from February 1, 2020, to May 13, 2020, and we identified three cases of acute STEMI who underwent coronary angiography. These patients diagnosed with COVID-19 had a positive result on polymerase chain reaction (PCR) testing of a nasopharyngeal sample. After being granted Institutional Review Board approval, subjects were identified by requesting access to a list of all hospitalized patients with COVID-19 from our Infection Control department.

## 3. Case 1

**Figure 1:**
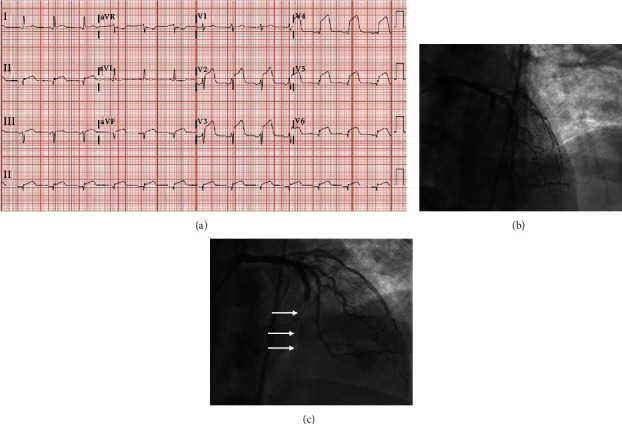
ECG on admission and LHC findings. (a) ECG showing ST elevations in anterior leads. (b) Initial coronary angiography. (c) Coronary angiography after PCI. Arrows indicate the course of LAD.

A 59-year-old Caucasian male was admitted for hypoxia in the setting of COVID-19. His past medical history was notable for type 2 diabetes mellitus, hyperlipidemia, and overweight BMI, and he took aspirin 81 mg daily for primary prevention. On admission, blood pressure (BP) was 148/79 mmHg, respiratory rate was 24, and oxygen saturation was 89% on room air. There were no notable findings on the physical exam. Chest X-ray (CXR) showed patchy bilateral pulmonary infiltrates. Hospitalization was complicated by acute chest pain on hospital day five with ECG showing anterior wall STEMI (Figure 1(a)). He was intubated and left heart catheterization (LHC) revealed 40% stenosis of distal left main artery (LM), 100% stenosis of midleft anterior descending artery (LAD), normal left circumflex (LCx), 80% stenosis of proximal right coronary artery (RCA), and proximal posterior descending artery (Figure 1(b)). The procedure was complicated by heavy thrombus burden in LAD and “no-reflow” phenomenon despite the use of rescue thrombectomy, multiple intracoronary vasodilators, and 3 drug-eluting stent (DES) placement; flow was not reestablished to the apex (Figure 1(c)), which was concerning for stent thrombosis. Troponin I peaked at 105.0 ng/mL (normal <0.015). Postcatheterization transthoracic echocardiogram (TTE) showed a left ventricular ejection fraction of 41-45% and distal anterior and apical akinesis. Treatment was started with ticagrelor, aspirin, atorvastatin, and metoprolol tartrate. Course was further complicated by elevated D-dimer of 33.35 mg/L FEU for which he was started on heparin drip, new atrial flutter, and septic shock in setting of prolonged ventilator requirement. He received digoxin 62.5 mcg daily, metoprolol tartrate 37.5 mg every 6 hours, and amiodarone loading dose followed by maintenance at 200 mg daily for atrial flutter. He eventually required the initiation of norepinephrine for blood pressure support, which also optimized the patient's heart rate, so metoprolol was discontinued. For management of COVID-19, the patient received a 5-day course of azithromycin, a 9-day course of hydroxychloroquine, one dose of tocilizumab, and a 5-day course of Lopinavir-Ritonavir, as was recommended by the Yale-New Haven Health system's treatment guidelines at the time. The patient expired on day 30 of hospitalization after the family opted to transition to comfort care.

## 4. Case 2

**Figure 2:**
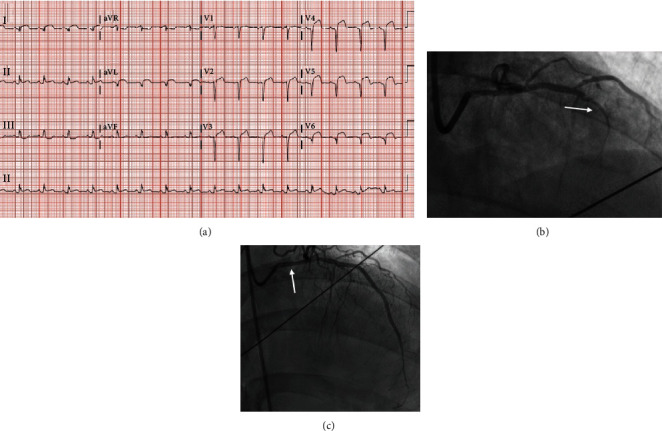
ECG on admission and LHC findings. (a) ECG showing ST elevations in anterolateral leads. (b) Initial coronary angiography with arrow showing occluded LAD. (c) Coronary angiography after PCI with arrow showing coronary vasospasm in distal LM.

A 47-year-old Hispanic male presented with a 2-week history of intermittent substernal chest pain with exertion and dyspnea. He had completed a 5-day course of azithromycin as an outpatient. His past medical history was notable for prediabetes, hypertension treated with amlodipine 5 mg daily, and class I obesity. Admission vital signs included BP 105/54 mmHg, pulse of 90 bpm, respiratory rate of 28, and oxygen saturation of 95% on room air. Physical exam was notable for bilateral rales. Labwork was pertinent for WBC of 15.3 ×1000/*μ*L, Troponin I of 72.4 ng/mL, pro-BNP of 1,184 pg/mL, and total CK of 3,549 U/L. COVID-19 PCR was positive. CXR showed mild heterogeneous bilateral ground-glass opacities. ECG demonstrated ST-elevation over anterolateral wall distribution (Figure 2(a)). The patient was electively intubated, and LHC revealed late presenting anterior wall MI with 30% stenosis of distal LM, 100% stenosis of LAD, normal LCx, and normal RCA. Left ventricular (LV) filling pressures were moderately elevated at 18 mmHg (Figure 2(b)). Coronary vasospasm was noted and was treated using intracoronary vasodilators. Two DES were placed in the proximal and distal LAD, but no reflow was observed with noteworthy thrombus burden likely from stent thrombosis (Figure 2(c)). Troponin I peaked at 131.2 ng/mL. Postprocedure TTE showed reduced LVEF of 35-40%, mild MR and TR, mild LVH, anterior, antero-apical akinesis-dyskinesis, and spontaneous echo-contrast in the LV apex. With rising D-dimer that peaked at 2.29 mg/L FEU, the patient was started on warfarin with enoxaparin bridge. The patient was extubated successfully one day after LHC, and he was started on metoprolol, atorvastatin, lisinopril, aspirin, and ticagrelor. He also required treatment with nitroglycerin drip for vasospasm. For management of COVID-19, he received a 5-day course of hydroxychloroquine. The patient was discharged home on room air after a 9-day hospitalization with recommendation to remain on oral anticoagulation for one month due to elevated D-dimer without any proven venous thromboembolism.

## 5. Case 3

**Figure 3:**
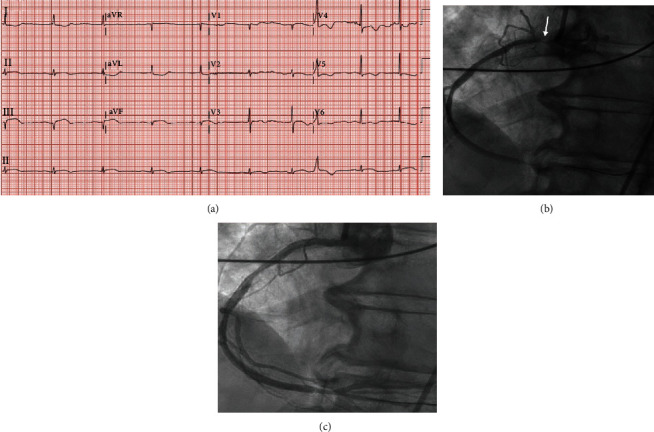
ECG on admission and LHC findings. (a) ECG showing ST elevations in inferolateral leads. (b) Initial coronary angiography with arrow showing RCA ostium. (c) Coronary angiography after PCI.

A 75-year-old African American female presented with acute nausea, diaphoresis, and weakness while climbing stairs. Past medical history was notable for hypertension treated with amlodipine 10 mg and hydrochlorothiazide 25 mg daily, overweight BMI, and illness four weeks prior to this admission that was suspected to be COVID-19. CXR showed diffuse interstitial changes. ECG performed by EMS showed atrial fibrillation with ST segment elevation. Vitals on presentation included HR 58 bpm, RR 22, BP 99/56mmHg, and SpO2 of 100% on room air. Physical exam was noncontributory. Laboratory work showed WBC 12.7 ×1000/*μ*L, D-dimer 0.6 mg/L, Troponin I <0.015 ng/mL (peaked 2.27 ng/mL), and positive COVID-19 PCR test. Repeat ECG in the ED demonstrated inferolateral ST-elevation (Figure 3(a)). Treatment of atrial fibrillation with rate-controlling medications was deferred to heart rate between 50 and 60 beats per minute and borderline low blood pressure. Coronary angiography revealed 99% ostial stenosis of the RCA with TIMI-2 flow, mid-RCA 40% stenosis, distal RCA 60% stenosis, 40% proximal lesion in LAD, and LCX without obstruction (Figure 3(b)). DES was placed to ostial RCA with good flow (Figure 3(c)). Post-PCI echocardiogram showed LVEF of 45-50%, basal inferior hypokinesis, mild to moderate MR, and left atrial enlargement. Systemic anticoagulation was not initiated due to the patient's spontaneous conversion to normal sinus rhythm after successful PCI.

The patient's hospital course was eventful for development of bilateral neck swelling and angioedema without airway compromise considered to be an atypical presentation of angioedema from ticagrelor use. These symptoms were treated with intravenous methylprednisone. The patient was discharged home after six-day hospitalization. She was comfortable on room air throughout this hospitalization, and Yale-New Haven Health system's COVID-19 treatment protocols did not recommend specific treatments for COVID-19 in patients who did not require supplemental oxygen.

## 6. Discussion

Key characteristics of each case varied including the timing of presentation, age, gender, race, and symptomology. However, all three patients had risk factors for CVD, including hypertension, hyperlipidemia, diabetes mellitus, and/or overweight or obese BMI. One patient expired during their hospitalization, and yet our two cases that presented with delayed symptom onset both survived. One subject displayed vasospasm in addition to coronary atherosclerotic obstruction and refractory thrombus formation. In addition, we noted signs of hypercoagulability such as flow not being reestablished fully in two cases as well as all three cases having elevated D-dimer, which is nonspecific but may signify that our patients were experiencing undetected thrombotic events or tendencies. None had prior history of venous thromboembolism, atrial fibrillation, or atrial flutter, so they were not on any oral anticoagulation therapy prior to hospitalization. Ultimately, two patients were started on anticoagulation due to new atrial flutter or significantly elevated D-dimer worrisome for venous thromboembolism. This highlights the need for clinicians to consider the role of oral anticoagulation started before or during hospital admission for COVID-19 patients. A recent retrospective study found that clinical outcomes were no better for COVID-19 patients who were previously on oral anticoagulation prior to hospital admission compared to those not on any anticoagulation, even after balancing for confounders such as differences in age and chronic disease burden [5]. While some of the most feared complications of COVID-19 are managed with anticoagulation, anticoagulation does not offer proven benefit for other serious complications of COVID-19 such as acute respiratory distress syndrome, and anticoagulation therapy carries risks. Therefore, we do not recommend therapeutic anticoagulation for COVID-19 patients in the absence of well-established indications.

A case series by Bangalore et al. reported a total of 18 cases of STEMI in patients with COVID-19 [6]. Of the 18 subjects, 9 underwent invasive intervention with angiography, but only two-thirds of those procedures confirmed obstructive coronary disease. That finding suggested that in addition to obstructive disease, there were nonobstructive cardiovascular diseases associated with COVID-19, which may represent perimyocarditis, ischemic/stress cardiomyopathy, or coronary vasospasm. There are numerous hypotheses regarding the mechanism of myocardial injury caused by SARS-CoV-2 infection. These include roles for (i) SARS-CoV-2 binding of angiotensin enzyme 2, which is found on myocytes and type 2 pneumocytes and which can directly cause toxicity to myocardial cells [7]; (ii) oxygen supply versus demand mismatch created secondary to hypoxemic respiratory failure arising from COVID-19-induced lung injury, which then causes type 2 myocardial infarction [8]; and (iii) COVID-19 induction of a prothrombotic effect related to cytokine-mediated inflammation and endotheliopathy, which can then lead to plaque instability, vasospasm, and rupture [9]. A recent case report describes the autopsy results of a patient with suspected STEMI and COVID-19, which found “extensive microvascular thrombosis in the absence of epicardial coronary obstruction despite no detectable virus in several sections of myocardial tissue [10]”.

The unprecedented nature of the COVID-19 pandemic justifies a reevaluation of standard of care for treating patients with STEMI. Recent commentary written by experts reveals an openness to debate the use of fibrinolytic therapy versus PCI as the primary intervention [11]. In a recent multicenter case series by Hamadeh et al., 21% of subjects with COVID-19 and STEMI treated with PCI experienced stent thrombosis, and overall in-hospital mortality risk was 12% [12]. An observational study by Choudry et al., which compared outcomes between STEMI patients with COVID-19 to those without the disease, also demonstrated increased thrombus burden in those who were infected [13]. This phenomenon correlated with what was observed in our group of STEMI patients. Given the evidence that COVID-19 patients with STEMI are at increased risk of stent thrombosis and potentially are more difficult revascularize, interventional strategies for these patients should be revisited in light of the fact that fibrinolytic therapy can be an effective treatment and exposes healthcare workers to lower theoretical risk of COVID-19 transmission.

There are several domains of investigation that are needed, including (i) determining the relative risk of STEMI and other myocardial injury in patients with COVID-19 compared to those without active infection, (ii) etiologies to explain any potential increase in relative risk so that focused therapies can be developed, and (iii) relative effectiveness of fibrinolytic therapy versus PCI, given that complications from COVID-19 may hamper the effectiveness of PCI.

## Data Availability

Figures used are available from the corresponding author on request.

## References

[1] Disease C. (2019). *(COVID-19) Weekly Epidemiological Situation Report*.

[2] Mahmud E., Dauerman H. L., FGP W. (2020). Management of Acute Myocardial Infarction During the COVID-19 Pandemic: A Position Statement From the Society for Cardiovascular Angiography and Interventions (SCAI), the American College of Cardiology (ACC), and the American College of Emergency Physicians (ACEP). *Journal of the American College of Cardiology*.

[3] Russo V., Bottino R., Carbone A. (2020). COVID-19 and heart: from clinical features to pharmacological implications. *Journal of Clinical Medicine*.

[4] Russo V., Rago A., Carbone A. (2020). Atrial fibrillation in COVID-19: from epidemiological association to pharmacological implications. *Journal of Cardiovascular Pharmacology*.

[5] Russo V., Bottino R., D’Andrea A. (2021). Chronic oral anticoagulation and clinical outcome in hospitalized COVID-19 patients. *Cardiovascular Drugs and Therapy*.

[6] Bangalore S., Sharma A., Slotwiner A. (2020). ST-segment elevation in patients with Covid-19 - a case series. *New England Journal of Medicine*.

[7] Hoffmann M., Kleine-Weber H., Schroeder S. (2020). SARS-CoV-2 cell entry depends on ACE2 and TMPRSS2 and is blocked by a clinically proven protease inhibitor. *Cell*.

[8] Castagna F., Cerrud-Rodriguez R., Villela M. A., Bortnick A. E. (2020). SARS-COV-2 infection presenting as ST-elevation myocardial infarction. *Catheterization and Cardiovascular Interventions*.

[9] Klok F. A., Kruip M. J. H. A., van der Meer N. J. M. (2020). Incidence of thrombotic complications in critically ill ICU patients with COVID-19. *Thrombosis Research*.

[10] Guagliumi G., Sonzogni A., Pescetelli I., Pellegrini D., Finn A. V. (2020). Microthrombi and ST-segment-elevation myocardial infarction in COVID-19. *Circulation*.

[11] Daniels M. J., Cohen M. G., Bavry A. A., Kumbhani D. J. (2020). Reperfusion of ST-segment-elevation myocardial infarction in the COVID-19 era: business as usual?. *Circulation*.

[12] Hamadeh A., Aldujeli A., Briedis K. (2020). Characteristics and Outcomes in Patients Presenting With COVID-19 and ST- Segment Elevation Myocardial Infarction. *The American Journal of Cardiology*.

[13] Choudry F. A., Hamshere S. M., Rathod K. S. (2020). High Thrombus burden in patients with COVID-19 presenting with ST-segment elevation myocardial infarction. *Journal of the American College of Cardiology*.

